# The Mode of Action of Isocyanide in Three Aquatic Organisms, *Balanus amphitrite*, *Bugula neritina* and *Danio rerio*

**DOI:** 10.1371/journal.pone.0045442

**Published:** 2012-09-18

**Authors:** Yi-Fan Zhang, Yoshikazu Kitano, Yasuyuki Nogata, Yu Zhang, Pei-Yuan Qian

**Affiliations:** 1 KAUST Global Collaborative Research Program, Division of Life Science, Hong Kong University of Science and Technology, Hong Kong SAR, China; 2 Laboratory of Bio-organic Chemistry, Tokyo University of Agriculture and Technology, Tokyo, Japan; 3 Abiko Research Laboratory, Central Research Institute of Electric Power Industry, Chiba, Japan; Shantou University Medical College, China

## Abstract

Isocyanide is a potential antifouling compound in marine environments. In this study, we investigated its mode of action in three aquatic organisms. Two of them, the bryozoan *Bugula neritina* and the barnacle *Balanus amphitrite*, are major marine fouling invertebrates, and the other organism is the non-target species zebrafish *Danio rerio*. In the swimming larvae of *B. neritina*, isocyanide did not affect the total attachment rate (≤50 µg ml^−1^), but it did change the attachment site by increasing the percentage of attachment on the bottom of the container rather than on the wall or air-water inter-surface. Isocyanide binds several proteins in *B. neritina* as identified via SDS-PAGE-LC-MS/MS: 1) a 30 kD protein band containing two proteins similar to voltage dependent anion channels (VDAC), which control the direct coupling of the mitochondrial matrix to the energy maintenance of the cytosol and the release of apoptogenic factors from mitochondria of mammalian cells; and 2) an unknown 39 kD protein. In *B. amphitrite* cyprids, the isocyanide binding protein were 1) a protein similar to NADH-ubiquinone oxidoreductase, which is the “entry enzyme” of oxidative phosphorylation in mitochondria; and 2) cytochrome P450. In *Danio rerio* embryos, isocyanide caused “wavy” notochords, hydrocephalus, pericardial edema, poor blood circulation, and defects in pigmentation and hematopoiesis, which phenocopied copper deficiency. This is the first report on isocyanide binding proteins in fouling organisms, as well as the first description of its phenotype and potential toxicology in zebrafish.

## Introduction

Biofouling leads to vast problems in marine industries and in the development of aquaculture. It involves the attachment by marine organisms to submerged surfaces, which increases the weight, drag and surface corrosion of ships and leads to huge maintenance costs in aquaculture systems and seawater pipelines [1]. It is estimated that without antifouling measures, the fuel consumption of ships would increase by up to 40% and the overall costs of a voyage would increase by as much as 77% [2]. To control biofouling, chemical compounds are mixed with paints that are applied to ships, pipes and other submerged surfaces to keep the painted areas clear of biofouling species. These chemical compounds are, however, often highly toxic to aquatic organisms [3], [4], [5] and many have been banned from use. There is an urgent demand on better and non- or less toxic antifouling compounds. To safeguard marine environments, it is really necessary to understand the environmental impacts and molecular targets of the new antifouling compounds in representative organisms before they are introduced to marine environment.

**Figure 1:**
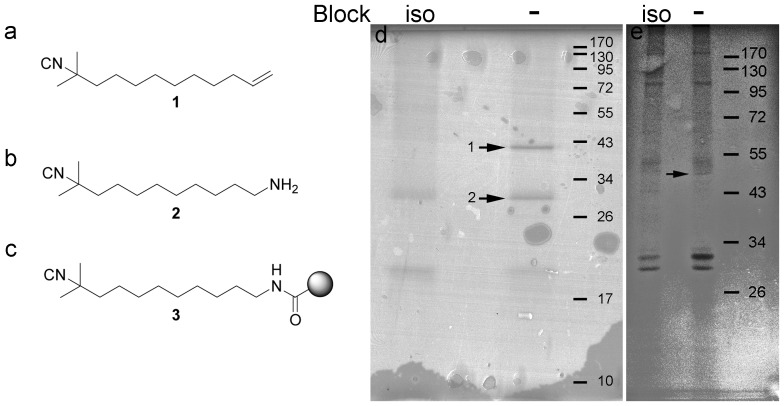
Chemical structures and affinity pull down assay results of isocyanide binding proteins. **a–c**) Chemical structure of a) isocyanide **1** [11-Isocyano-11-methyldodec-1-ene], b) isocyanide **2** [10-isocyano-10-methylundecan-1-amine], c) isocyanide **2** conjugated with the matrix (isocyanide-matrix). **d–e**) The coomassie blue G250 stained SDS-PAGE gel of SDS-sample-buffer-eluted proteins that were pulled down by the isocyanide-matrix. The lanes (Block: iso) contain proteins that were pulled down by the isocyanide-matrix in the presence of free isocyanide **2** in the binding solution, thus serving as the competition control for the (Block: -). d) Pulled down from the cell lysate of *Bugula neritina* swimming larvae. The identity of the protein bands was determined by UPLC-MS/MS (see also Table S1). e) Pulled down from the cell lysate of *Balanus amphitrite* cyprid. The 50 kD protein band (arrow) contains NADH-ubiquinone oxidoreductase and cytochrome P450 as identified by UPLC-MS/MS. The same cytochrome P450 also showed up in a lower non-specific band (43KD) in another batch of experiment as identified by LC-MS/MS (see also Table S2 and Figure S2).

Isocyanide **1** (11-Isocyano-11-methyldodec-1-ene) (Figure 1a) is a potential antifouling compound developed recently [6]. It was designed from an intensive study of structure-function relationship of a group of marine natural products [7] and could arrest biofouling by marine invertebrates in field tests [6]. In addition, it can be chemically synthesized on a large scale. Isocyanide **1** has a low EC_50_ (0.046 µg ml^−1^) and a high safety ratio (LC_50_/EC_50_>652) against a major fouling organism *Balanus amphitrite* [6]. However, the molecular targets of this compound remain unknown.

In this study, we report the possible molecular targets of isocyanide **1** in three aquatic organisms: the barnacle *B. amphitrite* and the bryozoan *Bugula neritina*, which are respectively the major hard fouling and soft fouling species that belong to distantly related taxonomic groups [8], [9]; and a non-target (non-biofouling) organism, zebrafish *Danio rerio*. We used affinity pull down assay to identify the isocyanide-binding proteins in the two target species since the same approach has been successfully used to identify the potential molecular targets of another antifouling compound butenolide (5-octylfuran-2(5H)-one, Figure S1) [10]. We chose *D. rerio*, simply because there is a large database of developmental defects (www.zfin.org) for this species. Therefore, the defects caused by small molecules can be linked to specific pathways known to cause the same defect [11], [12]. This “phenotype matching” approach has also been used successfully to identify the pro-apoptotic effect of butenolide [13]. One additional advantage of using the same approach to study isocyanide **1** and butenolide is that we can make a better comparison between these two potential antifouling compounds.

## Materials and Methods

### Chemicals

11-Isocyano-11-methyldodec-1-ene (referred to as isocyanide **1**, Figure 1a) and its analog 10-isocyano-10-methylundecan-1-amine (referred to as isocyanide **2**, Figure 1b) were synthesized according to published procedures [14].

### Animal Maintenance and Assays

*B. amphitrite* adults and cyprid larvae (competent to settle), as well as *B. neritina* adult colonies and swimming larvae (competent to settle) were obtained according to the methods described in previous studies [15], [16]. For the affinity pull down assays, the *B. amphitrite* cyprids and *B. neritina* swimming larvae were either immediately processed or stored in liquid nitrogen until further processing. For the *B. neritina* settlement assay, 30–70 swimming larvae per well were incubated in the dark in a 24-well plate. Various concentrations of the isocyanide **1** solution in filtered seawater (FSW) were used as the testing solutions. The numbers of larvae attached to the bottom of the well, to the wall of the well, and on water-air interface, as well as swimming larvae were counted under a dissection microscope after 48 h. Dead larvae (distinguished by a color change) were also counted.

*Danio rerio* (zebrafish) were bred as previously described [17]. Their embryos were kindly provided by Dr. Zilong Wen of Hong Kong University of Science and Technology. The toxicology assays were performed as previously described [13]. Nine isocyanide **1** concentrations were tested: 0, 0.5, 1.0, 1.5, 2.0, 2.5, 5, 10.0, and 20.0 µg ml^−1^. The embryos were kept in a humidified box at 28°C and were checked under a dissection microscope at the indicated time.

### Affinity Pull Down Assays

A previous study showed that both isocyanide **1** and **2** have antifouling activity, and their functional group is located in the isonitril structure [6]. Isocyanide **2** has a primary amine group at the end of its carbon chain far away from the isonitril group, which makes it suitable for covalent coupling to the Affi-gel® 10 (Bio-Rad, Hercules, CA, USA) matrix. We conjugated isocyanide **2** with the matrix according to Bio-Rad’s manual to make the isocyanide-matrix, with the isonitril group reaching out to proteins (Figure 1c). The isocyanide-matrix can be incubated with the solubilized cell lysate to pull down the isocyanide-binding proteins. In the competition control, isocyanide **2** was added to the cell lysate to reach a final concentration of 0.4 mg ml^−1^ before and during incubation with the isocyanide-matrix to block the protein-isocyanide-matrix interaction. After the incubation, the isocyanide-matrix was washed stringently with high ionic strength solutions (500 mM NaCl in TBS 1% Triton X100®). Subsequently, the proteins retained on the isocyanide-matrix were eluted by boiling in an SDS-sample buffer, separated by SDS/PAGE, visualized by Coomassie stain and analyzed by UPLC-MS/MS. The detailed affinity pull down protocol has been described in a recent publication from our laboratory, in which the potential molecular targets of another antifouling compound, butenolide, were identified [10].

All issues related to the animal ethics strictly followed the procedures established in Hong Kong University of Science and Technology. The work was approved by Committee on Research Practices of Hong Kong University of Science and Technology and strictly followed the university's established guideline.

**Figure 2:**
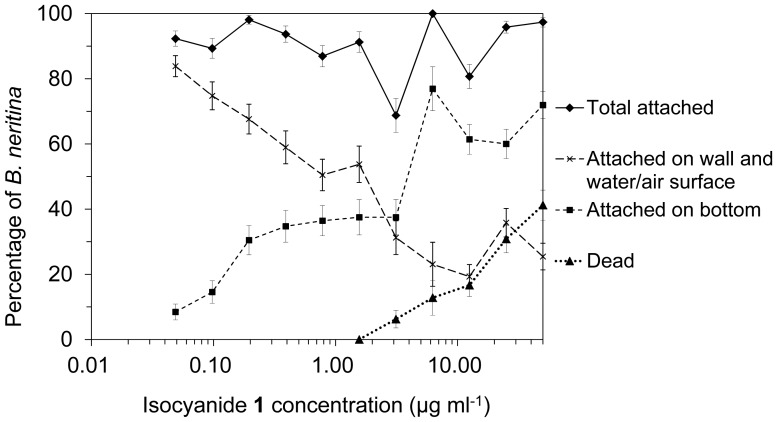
The effect of isocyanide 1 on the attachment and survival of *Bugula neritina* (observed at 48 h). Error bars represent standard errors. n ≥40 under each test condition.

## Results

### The Effect and Binding Proteins of Isocyanide in *B. neritina* Swimming Larvae

When treated with isocyanide **1**, *B. neritina* larvae changed their attachment site: more larvae attached to the bottom of the well, rather than to the wall or to the water-air interface as the control larvae preferred (Figure 2). Since most of the ship hull surfaces face downward to the water (at different degrees), the attachment site change induced by isocyanide **1** can protect these surfaces from fouling by *B. neritina*. The EC_50_ for the attachment site change was 1 µg ml^−1^, and the lowest observed effective concentration (LOEC) was 0.1 µg ml^−1^. The 48 h LC_50_ was more than 50 µg ml^−1^, whereas the 48 h LC_10_ was 4 µg ml^−1^. The LC_50_/EC_50_ ratio was more than 50. Most of the dead larvae had attached to the bottoms of the containers. Isocyanide **1** did not affect the total attachment rate of *B. neritina* at concentrations up to 50 µg ml^−1^.

A protein band with an apparent molecular weight of 30 kD was specifically pulled down from the cell lysate of *B. neritina* by isocyanide on the matrix (Figure 1d). Two proteins were identified from this 30 kD band; both were similar to voltage dependent anion channels (VDAC) (Table S1). Another isocyanide-specific protein band with an apparent molecular weight of 39 kD did not have significant hits in the Mascot search based on the current database.

### The Isocyanide Binding Proteins in *B. amphitrite* Cyprids

In *B. amphitrite*, an isocyanide-specific protein band with an apparent molecular mass of 50 kD was specifically pulled down by isocyanide on the matrix (Figure 1e). NADH-ubiquinone oxidoreductase was specifically identified in this protein band (Table S2). Cytochrome P450 (CYP) was also identified in this protein band with high mascot score consistently, but in one batch of experiments the same CYP protein sequence also appeared in a non-specific protein band with a lower apparent molecular weight (Figure S2); perhaps its partial degradation product overlapped with that non-specific band. In Interproscan, this CYP matches 7 out of 9 motifs (from motif 3 to 9) of E-class group I cytochrome P450 according to EP450I, which is a 9-element fingerprint that provides a signature for E-class group I P450s. In Blastp search, this CYP is similar to CYP15A1 in *Reticulitermes flavipes* (42% identity) and in *Tribolium castaneum* (38% identity). But the sequences determining their substrate binding sites are not exactly the same.

**Table 1:** Dose- and time-dependent effects of isocyanide 1 on zebrafish *Danio rerio* embryos.

Concentration of isocyanide 1	Number of zebrafish embryos											
		Eye pigmentation		Notochord		Head		Pericardial		Hematopoiesis		
µg ml^−1^	Total	Normal	Light	Straight	Wavy	Normal	Hydrocephalus	Normal	Edema	Normal	Deficient	Observation time
0	9	9	0	9	0	9	0	9	0	9	0	72 hpf
0.5	8	3	5	8	0	8	0	5	3	5	3	72 hpf
1	11	0	11	0	11	0	11	8	3	1	10	72 hpf
1.5	10	0	10	0	10	0	10	9	1	7	3	72 hpf
2	10	0	10	0	10	0	10	5	5	1	9	72 hpf
2.5	11	0	11	0	11	0	11	2	9	0	11	72 hpf
0	9	9	0	9	0	9	0	9	0	9	0	96 hpf
0.5	8	6	2	8	0	8	0	7	1	8	0	96 hpf
1	11	5	6	0	11	0	11	7	4	8	3	96 hpf
1.5	10	3	7	0	10	0	10	8	2	10	0	96 hpf
2	10	0	10	0	10	0	10	4	6	8	2	96 hpf
2.5	11	0	11	0	11	0	11	2	9	0	11	96 hpf

The treatment started within 2 hours post fertilization (hpf).

**Figure 3:**
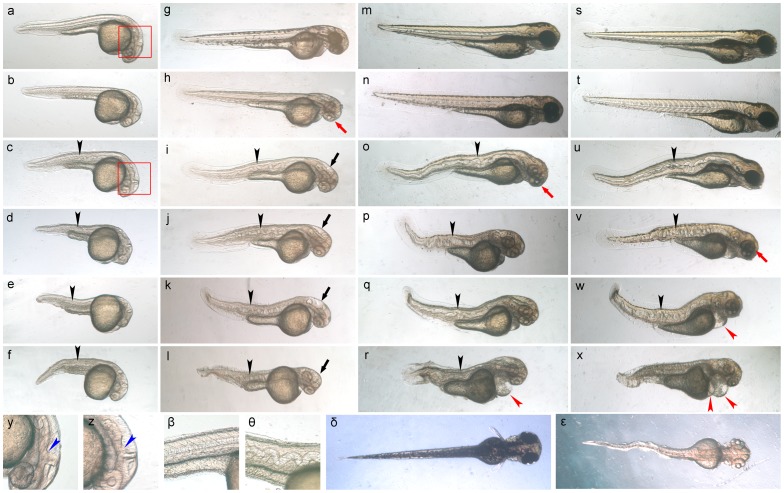
The effect of isocyanide 1 on *Danio rerio* (zebrafish) embryos. Zebrafish embryos were treated with isocyanide **1** at different concentrations from the early developmental stages (<2 hpf). **a–f**) Observed at 28 hpf. **g–l**) observed at 48 hpf; **m–r**) observed at 74 hpf; **s–x**) observed at 96 hpf; **a,g,m,s**) the control; **b,h,n,t**) 0.5 µg ml^−1^ isocyanide **1** treated; **c,i,o,u**) 1 µg ml^−1^ isocyanide **1** treated; **d,j,p,v**) 1.5 µg ml^−1^ isocyanide **1** treated; **e,k,q,w**) 2 µg ml^−1^ isocyanide **1** treated; **f,l,r,x**) 2.5 µg ml^−1^ isocyanide **1** treated. Note the “wavy” notochord (black arrowheads) and the hydrocephalus (black arrows) at concentrations ≥1 µg ml^−1^. Also note the pigmentation defect in the eyes (red arrows) and bodies of treated embryos. The red arrowheads point to pericardial edema, which developed under high concentrations. The rectangles in panels a and c indicate the area shown at higher magnification in panels y and z, respectively. **y,z**) High magnification of the embryo head at 28 hpf in the **y**) control and **z**) 1 µg ml^−1^ isocyanide **1**, revealing abnormal development in the isocyanide **1**-treated embryo (compare the structure indicated with blue arrowheads). **β,θ**) High magnification of the embryo notochord at 28 hpf in the **β**) control and **θ**) 1 µg ml^−1^ isocyanide **1**, revealing a wavy notochord in the isocyanide **1**-treated embryo. **δ,ε**) Top view of embryo at 72 hpf in the δ) control and ε) 1 µg ml^−1^ isocyanide **1**, revealing the lateral undulation of the notochords in the isocyanide **1**-treated embryo.

### The Effects of Isocyanide on *Danio rerio* Embryos

At 28 hours post fertilization (hpf), isocyanide **1**-treated zebrafish embryos (Figure 3c–f) showed notochord undulation and shorter bodies than those in the controls (Figure 3a). Their heads were also abnormal (Figure 3). At 48 hpf, the notochord undulation became more serious, with their bodies often bent or curved, and hydrocephalus became prominent (Figure 3i-l). These phenotypes were never corrected in later development. A pigmentation defect (especially in the eyes) became the most prominent phenotype at low concentrations of isocyanide **1** (Figure 3h and Table 1). At 74 hpf, embryos treated with low concentrations of isocyanide **1** started to catch up with normal embryos in their eye pigmentation (Figure 3n), while those treated with high concentrations of isocyanide **1** (Figure 3r) started to develop pericardial edema, and their blood volume was less than that in the control embryos. At 96 hpf, the embryos treated with low concentrations of isocyanide **1** (Figure 3t, u) continued to catch up with normal embryos in their eye pigmentation; but those treated with higher concentrations of isocyanide **1** continued development of pericardial edema and defective hematopoiesis (Figure 3w, x and Table 1). The phenotypes were saturated at isocyanide **1** concentrations greater than 5 µg ml^−1^ (Figure S3). The sensitivity order of these phenotypes was: pigment defect > notochord undulation & hydrocephalus >pericardial edema & hematopoiesis defect (Table 1). The phenotypes and the order of sensitivity were very similar to those of the copper deficiency phenotype caused by copper chelating molecules, which were also phenocopied by *calamity^vu69^* [18].

## Discussion

This is the first report on the protein binding of isocyanide in fouling organisms. *B. neritina* and *B. amphitrite* are species of choice because they are major biofouling organisms. The direct binding proteins of a drug are likely the drug’s molecular targets [19]. The isocyanide binding protein in *B. neritina* was found to be a homolog of VDAC. The VDAC family is located on the outer mitochondrial membrane. They control the direct coupling of the mitochondrial matrix to the energy maintenance of the cytosol and the release of apoptogenic factors from the mitochondria of mammalian cells [20]. Because of the essential roles of VDACs in cell metabolism and survival control, any alterations in their function would affect a cell’s physiology, which could easily cause changes in behaviors, such as the selection of an attachment site. The specific isocyanide binding protein in *B. amphitrite* was found to be a homolog of NADH-ubiquinone oxidoreductase, an enzyme located in the inner mitochondrial membrane that catalyzes the transfer of electrons from NADH to coenzyme Q. It is the "entry enzyme" for oxidative phosphorylation in the mitochondria (complex I) [21]. Because of the importance of this enzyme to cell respiration and metabolism, any alteration in its function caused by isocyanide binding could easily inhibit the activity of the larvae, including attachment and metamorphosis. Both VDAC and NADH-ubiquinone oxidoreductase are mitochondrial proteins, suggesting that isocyanide may influence mitochondrial functions. In addition, a cytochrome P450 (CYP) was consistently pulled down by isocyanide from *B. amphitrite* cell lysate, although it also appeared in a lower non-specific band in one of the other batches of experiment (Figure S2). The CYP systems are involved in drug metabolism and steroid hormone synthesis [22]. One of the most similar proteins, CYP15A1 in *Tribolium castaneum*, is involved in insect hormone biosynthesis and catalyzes the methyl farnesoate into juvenile hormone III (refer to KEGG pathway). So far the nature of the possible isocyanide-binding CYP and the nature of the interaction are still unknown.

**Table 2:** A comparison of biological effects between isocyanide 1 and butenolide.

	Species	Butenolide	Isocyanide 1
Inhibited species	Field test	invertebrates/diatom [23]	Invertebrates [6]
Effective concentrations (µg ml^−1^)	*Skeletonema costatum*	0.33 (5d IC_50_) [13]	NA f
	*Melita longidactyla*	3.02 (LC_50_) [13]	NA
	*Tigriopus japonicus*	2.56 (LC_50_) [13]	NA
	*Daphnia magna*	2.34 (EC_50_) [13]	NA
	*Lutjanus erythropterus*	1.32 (LC_50_) [13]	NA
	*Danio rerio*	0.89 (EC_50_) [13]	<0.5(EC_50_)
	*Balanus amphitrite*	0.518 (EC_50_) [23]	0.046 (EC_50_) [6]
	*Bugula neritina*	0.199 (EC_50_) [23]	0.1 (LOEC) g
	*Hydroides elegans*	0.0168 (EC_50_) [23]	NA
PNEC (µg ml^−1^) a		0.000168 [13]	NA
PEC b		NA	NA
Species selectivity ratio c		0.635 [13]	NA
Toxicology in non-target organism d	*Danio rerio*	apoptosis [13]	copper deficiency
Binding partners in target organisms e	General impression	affect primary metabolism for energygeneration [10]	affect mitochondrial functions
	*Balanus amphitrite*	ACAT1 (affect ketonebody synthesis) [10]	NADH-ubiquinone oxidoreductase (affect electron transport chain)and CYP
	*Bugula neritina*	ACADVL (affect fattyacid β-oxidation),actin and GSTs [10]	VDAC (affect mitochondrialenergy transport/apoptosis)
	*Vibrio sp.* UST020129-010	SCSβ (affect citricacid cycle) [10]	NA
metabolites/degradation products		NA	NA

a Predicted no effect concentration (PNEC) is calculated according to the guidelines for the testing of chemicals from the Organization for Economic Co-operation and Development (OECD) (http://www.oecd.org/dataoecd/6/14/2483645.pdf).

b Predicted environmental concentration (PEC).

c Species-selectivity ratio (based on acute toxicity test) = lowest L(E)C_50_ in non-target organisms/highest EC_50_ in target organisms determined preferably by settlement assay [13].

d Based on phenotype matching in zebrafish *Danio rerio.*

e Based on affinity pull down assay.

f NA: data not available.

g The lowest observed effective concentration (LOEC) to induce attachment site change in *Bugula neritina*. It should be noted that although the isocyanide **1** did not effectively inhibit the fouling of *Bugula neritina* in laboratory, it changed the settling site of this organism (Figure 2) and also effectively inhibited biofouling of *Bugula neritina* in field tests [6].

The affinity pull down is an effective and straight-forward approach to identify the binding partners and molecular targets of ligands, but it also has limitations. First, this technique is potentially limited by the accessibility of the functional group after immobilization to the insoluble matrix. To increase the accessibility of the matrix-conjugated compound to the proteins, we used the Affi-gel® 10 matrix which contains a neutral 10-atom spacer arm to hold the conjugates. Both isocyanide **1** (Figure 1a) [6] and butenolide (Figure S1) [23] have a linear structure with the functional group located on one end, which also makes their functional groups accessible after conjugating the other end to the matrix. Second, the disruptive nature of this technique may expose the ligand to components of certain cell compartments and environments that the ligand would not enter in vivo, leading to false-positive results. Complimentary approaches, such as *in vivo* imaging of the compound’s subcellular localization, could be considered to verify the results here. With the affinity pull down assay, we have successfully identified butenolide binding proteins, and their functional involvement as pharmaceutical targets can be supported by bioassays in living *B. amphitrite* and *B. neritina* [10]. Although there is no such a bioassay available for isocyanide-binding proteins because of their unique functions and the limited research models in marine organisms, proteins identified with affinity-pull-down assay are still the most likely molecular targets of isocyanide.

The zebrafish is a representative non-target organism of antifouling compounds. When treated with isocyanide **1**, the zebrafish embryos developed “wavy” notochords, hydrocephalus, and defects in eye pigmentation and hematopoiesis. These responses and the order of sensitivity were very similar to an embryo phenotype with copper-chelating-compound-induced copper deficiency, and the *calamity^vu69^* mutants whose phenotype was largely due to a nonfunctional mutation in the atp7a gene encoding copper transport ATPase [18]. The phenocopies suggested that isocyanide **1** might affect zebrafish embryos through the induction of copper deficiency. The “wavy” notochord is a characteristic defect in lysyl oxidase cuproenzymes, which crosslink collagens in the notochord sheath and need copper [24], [25], [26]. The defect in melanin pigmentation in the copper deficiency and *calamity^vu69^* mutants was probably due to the blocking of tyrosinase (a copper-containing oxidase) activity [18], [27]. The similarity in their phenotypes suggests that lysyl oxidase cuproenzymes, chondrocyte development and tyrosinase may be the target enzymes/processes of isocyanide **1** in zebrafish although no protein interactions were observed in this study.

We compared the biological effect of isocyanide **1** with butenolide in Table 2. There are still some missing points for future studies. Most importantly, for any new commercial antifouling compound, the predicted no effect concentration (PNEC) should be higher than the predicted environmental concentration (PEC) both inside harbors and in shipping lanes [28]. In addition, as we proposed in Zhang et al [13], the “species-selectivity ratio” should also be used to evaluate the environmental risk of antifouling compounds under the most extreme conditions, which are most likely to exist in places near the antifouling coatings (see the footnote of Table 2 for the calculation formula). A systematic toxicity profile of isocyanide **1** is needed to calculate the PNEC and “species-selectivity ratio”. The PECs of the two compounds should be investigated in the future. In addition, the metabolites and degradation products of these compounds should also be characterized and compared as they may also have biological activity.

## Supporting Information

Figure S1 **The structure of another antifouling compound butenolide.**
(TIF)

Figure S2 **Another affinity pull down assay result of isocyanide binding proteins in** ***Balanus amphitrite*** **cyprid cell lysate (see also** **Figure 1e** **).** The SDS-PAGE gel was stained with Coomassie Blue G250. The lanes (Block: iso) contain proteins that were pulled down by the isocyanide-matrix in the presence of free isocyanide **2** in the binding solution, thus serving as the competition control for the (Block: -). Protein band 1 corresponds to the protein band indicated by arrow in Figure 1e. Protein band 2 is absent in Figure 1e. Both NADH-ubiquinone oxidoreductase (isotig11604_32) and cytochrome P450 (isotig18086_24) can be identified in protein band 1 consistently, but cytochrome P450 can also be identified in protein band 2 in this batch of experiment.
(TIF)

Figure S3 **The effect of isocyanide 1 at high concentrations on** ***Danio rerio*** **(zebrafish) embryos.** Zebrafish embryos were treated with isocyanide **1** at different concentrations from the 2-cell stage. **a–f**) Observed at 24 hpf. **g–l**) Observed at 50 hpf. **m–r**) Observed at 76 hpf. **a,g,m**) the control; **b,h,n**) 1 µg ml^−1^ isocyanide **1** treated; **c,i,o**) 2.5 µg ml^−1^ isocyanide **1** treated; **d,j,p**) 5 µg ml^−1^ isocyanide **1** treated; **e,k,q**) 10 µg ml^−1^ isocyanide **1** treated; **f,l,r**) 20 µg ml^−1^ isocyanide **1** treated. The black arrowheads point to the “wavy” notochord; the black arrows indicate the hydrocephalus in treated embryos; the red arrowheads point to the pericardial edema; the red arrows indicate the congestion below the heart area. Note that the phenotypes were saturated at isocyanide **1** concentrations greater than 5 µg ml^−1^.
(TIF)

Table S1 **UPLC-MS/MS result for isocyanide binding proteins in** ***Bugula neritina*** **.** Related to Figure 1d.
(XLS)

Table S2 **UPLC-MS/MS result for isocyanide binding proteins in** ***Balanus amphitrite.*** Related to Figure 1e.
(XLS)
