# Supplementary figures and images for: The Mode of Action of Isocyanide in Three Aquatic Organisms, Balanus amphitrite, Bugula neritina and Danio rerio
Source: PLoS One. 2012 Sep 18;7(9):e45442. doi: 10.1371/journal.pone.0045442 (PMC3445549; doi:10.1371/journal.pone.0045442)

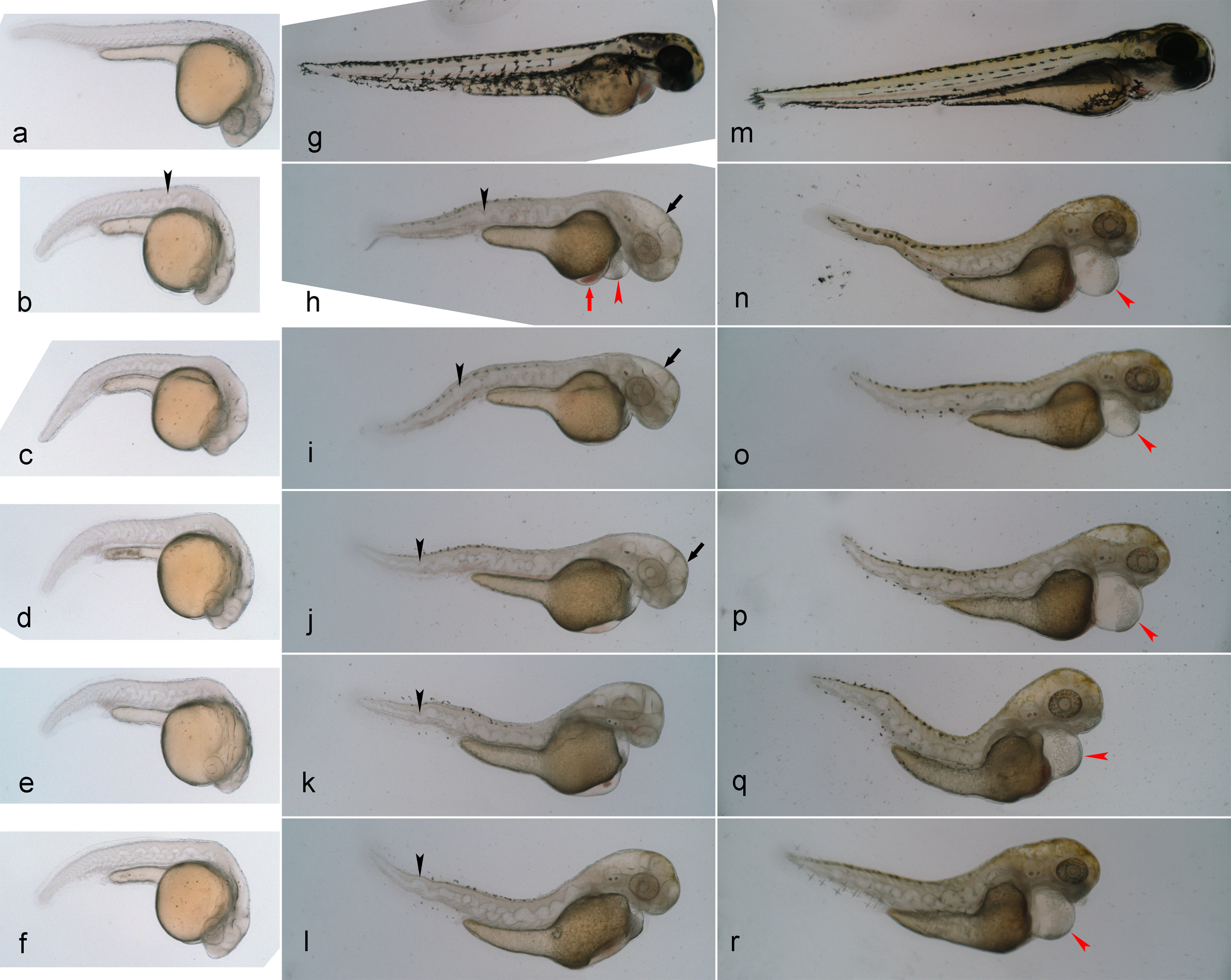

Supplement: Figure S3 — The effect of isocyanide 1 at high concentrations on Danio rerio (zebrafish) embryos. Zebrafish embryos were treated with isocyanide 1 at different concentrations from the 2-cell stage. a–f) Observed at 24 hpf. g–l) Observed at 50 hpf. m–r) Observed at 76 hpf. a,g,m) the control; b,h,n) 1 µg ml−1 isocyanide 1 treated; c,i,o) 2.5 µg ml−1 isocyanide 1 treated; d,j,p) 5 µg ml−1 isocyanide 1 treated; e,k,q) 10 µg ml−1 isocyanide 1 treated; f,l,r) 20 µg ml−1 isocyanide 1 treated. The black arrowheads point to the “wavy” notochord; the black arrows indicate the hydrocephalus in treated embryos; the red arrowheads point to the pericardial edema; the red arrows indicate the congestion below the heart area. Note that the phenotypes were saturated at isocyanide 1 concentrations greater than 5 µg ml−1. (TIF) [file pone.0045442.s003.tif]
